# The unusual cytoarchitecture of “vitelline follicles” in freshwater blood flukes of the genus *Sanguinicola* (Digenea, Aporocotylidae)

**DOI:** 10.1051/parasite/2021070

**Published:** 2021-10-26

**Authors:** Larisa G. Poddubnaya, Alexander Zhokhov, David I. Gibson

**Affiliations:** 1 Institute for Biology of Inland Waters, Russian Academy of Sciences 152742 Borok Yaroslavl Province Russia; 2 Department of Life Sciences, Natural History Museum Cromwell Road SW7 5BD London United Kingdom

**Keywords:** Vitelline cells, Intercellular junctions, Vitelline duct, Evolutionary lineage

## Abstract

This is the first study assessing the cytoarchitecture of the vitellarium of members of the freshwater, teleost-infecting lineage of blood-flukes (Aporocotylidae). The vitelline cytoarchitecture of two innominate species of *Sanguinicola* from freshwater fishes in Russia showed that vitelline cells at different stages of maturation are widely distributed throughout much of the body and are mixed with other cell types. The latter feature indicates that use of the term “follicular vitellarium” is inappropriate for species of this genus. An additional characteristic of the vitelline cells in these *Sanguinicola* spp. is their ability to form long, pseudopodia-like extensions of the peripheral cytoplasm that contact neighbouring vitelline cells and sarcoplasmic extensions, forming both heterologous and homologous intercellular junctions. Within the vitelline duct lumen, the cytoplasm of mature vitelline cells is filled with regular clusters (0.5–1.0 μm in diameter), comprising 10–30 vitelline globules, which have heterogeneous contents and electron-lucent lipid droplets (1.1–1.7 μm in diameter), but no apparent modifications of vitelline globules occur within the vitelline duct. The flattened, ciliated, epithelial lining of the common vitelline duct contains intra-epithelial nuclei, its luminal surface bears shallow lamellae and adjacent cells are adjoined by apical septate junctions. All of these observations, when compared to the marine *Aporocotyle simplex*, likely represent additional characteristics supporting the divergent evolutionary lineages of marine and freshwater aporocotylids.

## Abstract (fr)

Il s’agit de la première étude évaluant la cytoarchitecture du vitellarium des membres de la lignée des digènes (Aporocotylidae) infectant le sang des téléostéens d’eau douce. La cytoarchitecture vitelline de deux espèces non nommées de *Sanguinicola* de poissons d’eau douce de Russie a montré que les cellules vitellines à différents stades de maturation sont largement distribuées dans une grande partie du corps et sont mélangées avec d’autres types cellulaires. Cette dernière caractéristique indique que l’utilisation du terme « vitellarium folliculaire » est inappropriée pour les espèces de ce genre. Une caractéristique supplémentaire des cellules vitellines de ces *Sanguinicola* spp. est leur capacité à former de longues extensions du cytoplasme périphérique ressemblant à des pseudopodes, qui entrent en contact avec les cellules vitellines voisines et les extensions sarcoplasmiques, formant des jonctions intercellulaires à la fois hétérologues et homologues. Dans la lumière du canal vitellin, le cytoplasme des cellules vitellines mûres est rempli de grappes régulières (0,5 à 1,0 μm de diamètre), comprenant 10 à 30 globules vitellins, qui ont des contenus hétérogènes et des gouttelettes lipidiques (1,1 à 1,7 μm de diamètre) transparentes aux électrons, mais aucune modification apparente des globules vitellines ne se produit dans le canal vitellin. Le revêtement épithélial aplati et cilié du canal vitellin commun contient des noyaux intra-épithéliaux, sa surface luminale porte des lamelles peu profondes et les cellules adjacentes sont jointes par des jonctions septées apicales. Toutes ces observations, comparées à *Aporocotyle simplex* qui est marin, représentent probablement des caractéristiques supplémentaires soutenant que les lignées évolutives des Aporocotylidae marins et d’eau douce sont divergentes.

## Introduction

In the Digenea, the vitellarium is morphologically diverse; variations include single or double compact masses, a range of lobed or tubular structures and few to numerous isolated or linked follicles. Its configuration has been used as an important taxonomic or even phylogenetic criterion at several levels [4]. Furthermore, judging from the existing literature on ultrastructure, some characteristics of the vitelline cytoarchitecture have been used as discriminatory traits for digenean lineages [24, 25]. The latter traits include the presence of one or two types of cell components within the vitellarium, i.e., vitelline cells (vitellocytes) at different stages of development and interstitial cells; the occurrence of junctional complexes between cells within the vitellarium; and the presence or absence of a specialized sheath of the basal matrix, which isolates the vitellarium from the surrounding cells and organs. Most of such ultrastructural studies on digenean species have targeted the process of vitellogenesis, but, in a number of studies, the cytoarchitecture of the vitellarium itself has been investigated [6–8, 11–13, 24, 25, 30, 35, 36].

It is challenging to understand the evolution of the Digenea without detailed studies on their early branching groups. Although tree topologies fluctuate, depending on the number and identity of taxa included in the analysis, the fish blood flukes are typically and routinely recovered as an early-branching monophyletic group within the Digenea [21, 23]. Species of this family, especially the freshwater members/lineages, have been little studied using the transmission electron microscope (TEM) [18–20, 28, 29]. The phylogenetic relationships between the three postulated aporocotylid evolutionary lineages (chondrichthyan-infecting, freshwater teleost-infecting and marine teleost-infecting) [1] are only partly supported by morphology and genetics [1, 21–23].

There is only one detailed ultrastructural study on the cytoarchitecture of the vitellarium of an aporocotylid. This concerns the marine, teleost-infecting species *Aporocotyle simplex* Odhner, 1900 [27]. The present study of blood flukes of the genus *Sanguinicola* Plehn, 1905 is the first TEM investigation of the vitellarium of members of the freshwater, teleost-infecting lineage. Within this study, we will examine the phylogenetic relationships between freshwater and marine aporocotylids from teleosts based on the ultrastructural characteristics of their vitellarium.

## Materials and methods

### Ethics statement

All necessary permits for fishing were obtained for the authors by the Institute for Biology of Inland Waters, Russian Academy of Sciences, from the relevant authorities of the Russian Federation. The treatment of the collected fishes and the investigations of *Sanguinicola* spp. infections are in compliance with all institutional, national, and international guidelines on the care and use of animals.

### Specimens and species

**Figure 1:**
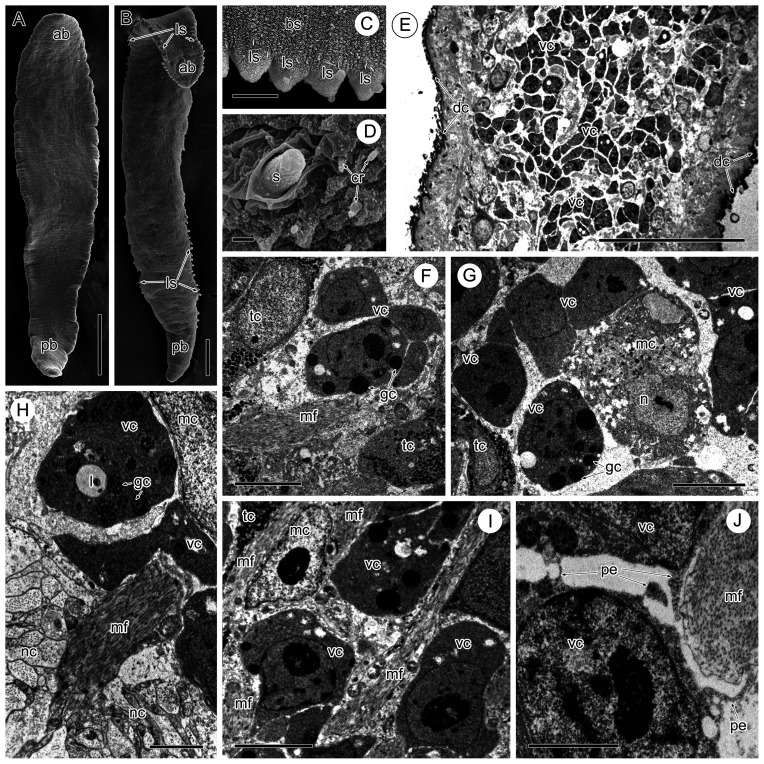
SEM images of *Sanguinicola* spp. (A–D) and TEM images of the vitelline cells of *Sanguinicola* sp. 1 from pike (E–J). (A) *Sanguinicola* sp. 1 from pike, note the absence of visible lateral spines along the body. Scale bar = 200 μm. (B) *Sanguinicola* sp. 2 from ides, note the presence of lateral spines along the body surface. Scale bar = 100 μm. (C) Lateral spines of *Sanguinicola* sp. 2. Scale bar = 10 μm. (D) Spine of *Sanguinicola* sp. 1 localized laterally in the anterior third of the body. Scale bar = 1 μm. (E) Longitudinal section through part of the body, note vitelline cells at different stages of maturation mixed with other cell types. Scale bar = 50 μm. (F) Vitelline cells surrounded by tegumentary cells and muscle fibres. Scale bar = 5 μm. (G) Vitelline cells mixed with muscle and tegumental cells. Scale bar = 5 μm. (H) Vitelline cells surrounded by a muscle cell, note the neighbouring nerve cord and muscle fibres. Scale bar = 2 μm. (I) Mixed vitelline cells, muscle cells and muscle fibres. Scale bar = 5 μm. (J) Pseudopodia-like extensions of a vitelline cell directed towards a neighbouring vitelline cell and muscle fibres. Scale bar = 2 μm. Abbreviations: ab = anterior part of body, bs = body surface, cr = ciliary receptor, dc = distal cytoplasm of tegument, gc = globular cluster, l = lipid droplet, ls = lateral spine, mc = muscle cell, mf = muscle fibres, n = nucleus, nc = nerve cord, pb = posterior part of body, pe = pseudopodium-like extension, s = spine, tc = tegumentary cell, vc = vitelline cell.

Specimens of *Sanguinicola* sp. 1 were collected from the ventral aorta leading from the heart of naturally infected pike *Esox lucius* (Linnaeus, 1758) (Esocidae) and specimens of *Sanguinicola* sp. 2. were collected from the ventral aorta of the heart of naturally infected ides *Leuciscus idus* (Linnaeus, 1758) (Cyprinidae) from the Rybinsk Reservoir in the Upper Volga River Basin, Russia. Using scanning electron microscopy (SEM), these two *Sanguinicola* spp. have been shown to differ morphologically (Figs. 1A–1D).

The status of species of *Sanguinicola* in European waters is in need of revision. This is especially so for the type-species, *S. inermis* Plehn, 1905, which has been reported from a wide range of hosts but especially from cyprinids. In this work, we are treating the two species studied as innominate, pending a detailed taxonomic study. *Sanguinicola* sp. 1 is the same species as that previously referred to as *Sanguinicola inermis* [28] and *Sanguinicola* sp. [29].

### Electron microscopy

For electron microscopy, live blood flukes were fixed using 3% glutaraldehyde in a 0.1 M sodium cacodylate buffer (pH 7.2) for 7 days at 5 °C, rinsed three times for 10 min periods in the same buffer and postfixed in 1% osmium tetroxide for 1 h. For SEM observations, fixed specimens were dehydrated in a graded ethanol series, with a final change to absolute acetone and then critical-point dried with liquid CO_2_. Later, the specimens were mounted on stubs, sputter-coated with gold-palladium and examined under a JEOL-JSM-6510LV microscope operating at 30 kV. For transmission electron microscopy (TEM), fixed specimens were later embedded in a mixture of Araldite and Epon using an Araldite/Embed-812 EM Embedding kit (EMS). Ultrathin sections were then stained with uranyl acetate and lead citrate, and examined using a JEM 1011 microscope operating at 80 kV.

## Results

**Figure 2:**
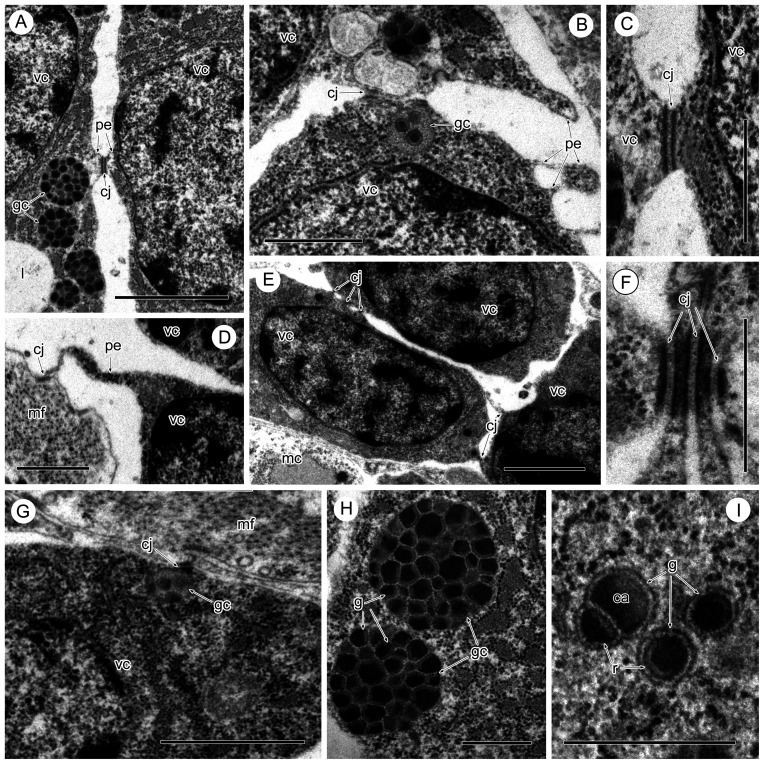
Intercellular junctions of the vitelline cells in *Sanguinicola* sp. 1 from pike. (A) Between two maturing vitelline cells, note the short cytoplasmic extension of both cells at the contact point. Scale bar = 2 μm. (B) Between two vitelline cells at an early stage of maturation, note the different kinds of pseudopodia-like extensions of the surface cytoplasm. Scale bar = 1 μm. (C, F) Detail of the junctions. Scale bars = 0.5 μm. (D) Between an immature vitelline cell and muscle fibres, note the long cytoplasmic extension of the vitelline cell at the contact point. Scale bar = 1 μm. (E) Between immature vitelline cells. Scale bar = 2 μm. (G) Between maturing vitelline cell and muscle fibres. Scale bar = 1 μm. (H) Vitelline clusters filled with vitelline globules. Scale bar = 1 μm. (I) Heterogeneous contents of the vitelline globules. Scale bar = 0.5 μm. Abbreviations: ca = central area of vitelline globule, cj = cell junction, g = vitelline globule, gc = globular cluster, mc = muscle cell, mf = muscle fibres, pe = pseudopodia-like extension, r = lucid rim of vitelline globule, vc = vitelline cell.

**Figure 3:**
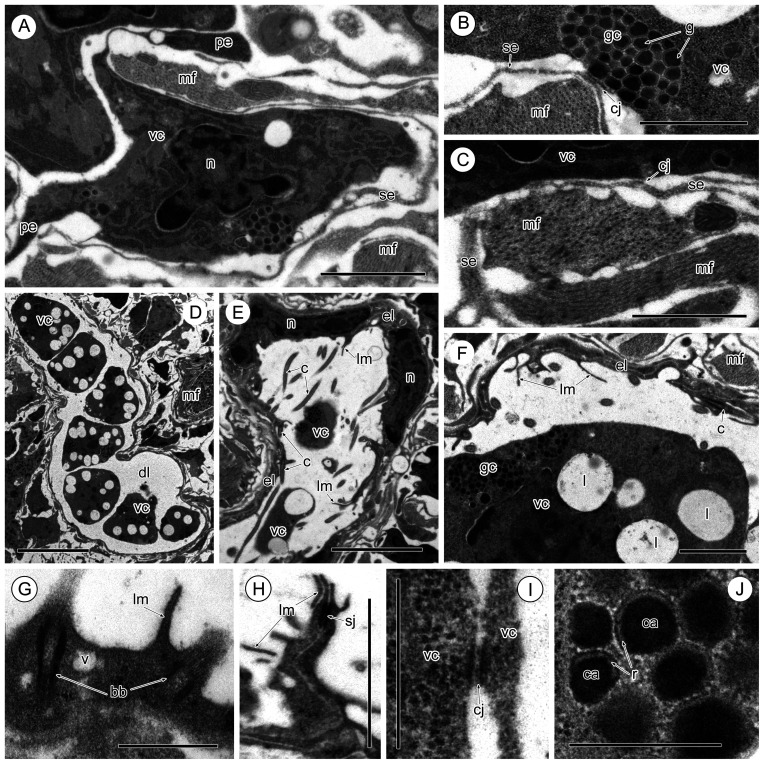
Vitelline cells (A–C, I, J) and vitelline duct (D–H) of *Sanguinicola* sp. 2 from ides. (A) Vitelline cell showing the various shapes of the cytoplasmic extensions, note the presence of numerous muscle fibres around this cell. Scale bar = 2 μm. (B) Intercellular junction between a vitelline cell and the thin, sarcoplasmic extension of a muscle cell. Scale bar = 1 μm. (C) Cell junction between a vitelline cell and a sarcoplasmic extension. Scale bar = 1 μm. (D) Part of the vitelline duct filled with mature vitelline cells. Scale bar = 10 μm. (E, F) Flattened, ciliated, epithelial lining of the vitelline duct showing the inter-epithelial nuclei of these cells. Scale bars = 5 μm (E) and 2 μm (F). (G) Region of the epithelial lining of the vitelline duct showing basal ciliary bodies and a short surface lamella. Scale bar = 0.5 μm. (H) Apical septate junction in the duct epithelium. Scale bar = 0.5 μm. (I) Intercellular junction between two vitelline cells. Scale bar = 0.5 μm. (J) Heterogeneous vitelline globules. Scale bar = 0.2 μm. Abbreviations: bb = basal body of cilium, c = cilium, ca = central area of vitelline globule, cj = cell junction, dl = vitelline duct lumen, el = epithelial lining of vitelline duct, g = vitelline globule; gc = globular cluster, l = lipid droplet, lm = lamella, mf = muscle fibres, n = nucleus, pe = pseudopodium-like extension, r = lucid rim of vitelline globule, se = sarcoplasmic extension, sj = septate junction, v = vesicle, vc = vitelline cell.

Low magnification TEM sections throughout the body of both species show the presence of numerous isolated vitelline cells at various stages of development; these are mixed with other types of cells (Fig. 1E). Along with muscle cells and their extensions, which dominate, in the immediate vicinity of vitelline cells there are tegumentary cells and nerve cords (Figs. 1F–1I, 3A–3C). These vitelline cells are either loosely associated with each other (Fig. 1I) or occur in small gatherings of 3–5 cells which are more closely associated (Figs. 1F, 1G, 2E).

Maturing and mature vitelline cells are characterized by the presence within their cytoplasm of clusters of vitelline globules and lipid droplets (Figs. 1G–1H, 2A, 2H, 3F). In sections, a cluster comprises 10–30 vitelline globules, which are round, membrane-bound structures (Fig. 2H). Depending on the number of globules in each cluster, its diameter can vary from 0.5 to 1.0 μm, but, on rare occasions, individual clusters may reach 1.7 μm in diameter. Individual vitelline globules present within the cluster measure 0.1–0.2 μm in diameter, and the space between the globules is filled with a finely granular material of moderate electron density (Figs. 2B, 2H). The heterogeneous contents of the globules include an electron-dense central area surrounded by a thin, more lucid rim (Figs. 2I, 3J). The lipid droplets (1.1–1.7 μm in diameter) within the vitelline cells contain an electron-lucent material with occasional patches of loosely packed material of medium density (Figs. 1H, 3F).

Most vitelline cells at different stages of their development are irregular in outline and have short or long pseudopodia-like extensions of the peripheral cytoplasm (Figs. 1J, 2A, 2B, 2D, 3A, 3B). Usually, the limiting plasma membranes of vitelline processes are close to neighbouring vitelline cells or sarcoplasmic processes (Figs. 1J, 2A, 2B, 2D, 2E, 2G, 3B, 3C). In such places, intercellular junctions resembling *zonulae adherens* are present between adjacent plasma membranes (Figs. 2A–2G, 3B, 3I).

The common vitelline duct of *Sanguinicola* spp. runs medially along the body and is lined by a flattened, ciliated epithelium (Figs. 3D–3F), whose intra-epithelial nuclei are flat, large and contain scattered areas of heterochromatin (Fig. 3E). The luminal surface of each epithelial cell is protuberant, forming shallow lamellae (Figs. 3E–3H), and its cytoplasm contains ciliary basal bodies, numerous free ribosomes, a few electron-lucent vesicles and mitochondria (Fig. 3G). Cilia are unevenly distributed both singly and as closely associated pairs on the luminal surface (Figs. 3E–3G). Adjacent cells are adjoined by apical septate junctions (Fig. 3H). Sparse muscle fibres are found beneath the duct epithelium (Figs. 3D, 3F). Isolated, mature vitelline cells are present within the lumen of the duct, aligned in a single row along its entire length (Figs. 3D, 3F). Their cytoplasm is filled with regular clusters of globules and lipid droplets (Figs. 3D, 3F). No apparent modifications of these vitelline globules were observed.

## Discussion

The present study has revealed some unusual features in the cytoarchitecture of the vitellarium of two innominate species of *Sanguinicola* (from pike *Esox lucius* and ide *Leuciscus idus*). An unusual characteristic of the vitellarium in these species is the presence of single vitelline cells, or a gathering of a small number of vitelline cells, at different stages in their development, which are widely distributed throughout much of the body and mixed with other cell types rather than comprising follicles. Consequently, the term vitelline “follicle” should not be used for these, and probably other, species *Sanguinicola*. Traditionally, the term “follicular vitellarium” in digeneans has been used to describe the accumulation of vitelline cells at different stages of maturation, situated close together and formed into small, usually numerous, compact vitelline masses. In the present material, each follicle contains only a single cell type, i.e., vitelline cells, which are not isolated from the surrounding tissue. The cytoarchitecture of vitelline follicles proper has previously been described for another aporocotylid, *Aporocotyle simplex*, which belongs to the marine, teleost-infecting aporocotylid lineage [27]. Vitelline follicles with the usual cytoarchitecture have also been described in digeneans of the orders Diplostomida, i.e., the diplostomatid *Pharyngostomoides procyonis* [5], and Plagiorchiida, i.e., the microphallids *Maritrema linguilla* [11] and *M. feliuli* [35], the cryptogonimids *Aphallus tubarium* [6] and *Metadena depressa* [7], the pleurogenid *Brandesia turgida*, [25] and the opecoelid *Cainocreadium labracis* [36]. However, in the gorgoderids *Gorgoderina vitelliloba* [12] and *Phyllodistomum angulatum* [24], the fasciolid *Fasciola hepatica* [10, 13], the gyrabascid *Allassogonoporus amphoraeformis* [30] and the azygiid *Azygia lucii* [24], a different kind of vitelline cytoarchitecture has been indicated, where both vitelline and interstitial cells are found surrounded by a basal matrix. In the case of the blood flukes, i.e., members of the superfamily Schistosomatoidea, which includes the Aporocotylidae, Spirorchiidae and Schistosomatidae, apart from the abovementioned aporocotylids, *A. simplex* [27] and two *Sanguinicola* spp. (present study), the ultrastructure of proper vitelline follicles has been studied in *Schistosoma* spp. [2, 3, 15]. In the Aspidogastrea, the sister group to the Digenea [21, 23], observations on *Aspidogaster limacoides* (Aspidogastridae, Aspidogastrinae) [17, 26], *Rohdella amazonica* (Aspidogastridae, Rohdellinae) [37] and *Rugogaster hydrolagi* (Rugogastridae) [31] indicate different vitelline patterns. The vitelline follicles of the rugogastrid *R. hydrologi* are isolated and surrounded by a “basal lamina and fibrous matrix” [31], whereas in the aspidogastrid *A. limacoides* there is no special limiting basal matrix and sarcoplasmic extensions of muscle cells penetrate deep into the follicles [26]. Based on the limited available data and the above examples of the vitelline ultrastructure in members of the Trematoda, it may be that the cytoarchitecture of the vitellarium could prove useful as an additional characteristic feature at family and lower levels. Evidence for this comes from the family Microphallidae, where the species studied both possess the same level of vitelline organization [11, 35]. However, variation in the vitelline cytoarchitecture of studied members of the Aporocotylidae ([27], present study) may reflect a lower (than family) level affiliation with the different lineages within the Aporocotylidae, as outlined in molecular studies [1].

An additional feature of the vitelline cells of *Sanguinicola* spp. is their ability to form long pseudopodia-like extensions of the peripheral cytoplasm, the limiting plasma membrane of which may be in contact with neighbouring vitelline cells or their sarcoplasmic extensions. Both heterologous (i.e., those occurring between adjacent membranes of vitelline cells and the sarcoplasmic processes of the muscle cells) and homologous (i.e., those occurring between adjacent membranes of vitelline cells) intercellular junctions have been shown to be present in the two *Sanguinicola* spp. studied. Interestingly, these intercellular junctions have been observed between vitelline cells at the same stage and at different stages of maturation. There are previous reports of the presence of heterologous and homologous contact sites between the vitelline follicles, i.e., in the monopisthocotylean monogenean *Ancyrocephalus paradoxus* [26], the aspidogastrid aspidogastrean *Aspidogaster limacoides* [26] and digeneans of the families Diplostomatidae [5], Microphallidae [11, 35], Pleurogenidae [25] and Aporocotylidae ([27], present study). The presence of intercellular junctions in digeneans possessing a proper follicular vitellarium may suggest a close relationship between vitelline cells and between vitelline and muscle cells. Despite the visible isolation of vitelline cells in freshwater aporocotylids indicated above, they maintain their intercellular interconnections due to their ability to form cytoplasmic pseudopodia-like extensions.

The present study has shown that the dominant inclusions in mature vitelline cells in both species of *Sanguinicola* studied herein, as in the marine *Aporocotyle simplex* [27], are clusters of vitelline globules. Individual vitelline globules of these aporocotylids have a heterogeneous matrix possessing central and peripheral areas of greater density (Present study, [27]). Judging from the available literature on the morphology of vitelline globules in the Digenea, similar globules have not been found in other studied taxa [2, 9–13, 24, 35, 36]. In the studied aporocotylids*,* the clusters are small, with a diameter of 0.5–1.0 μm, and contain 10–30 globules in the case of *Sanguinicola* spp. and 0.8–1.8 μm with 10–20 globules in *A. simplex* (Present study, [27]). Differences in the morphology, size and number of digenean vitelline clusters and globules within the clusters may be related to variations in the structure, size and number of the eggs and the size of the eggshell [3, 11, 24, 25, 34–36]. Vitelline globules represent building material for eggshell formation [34], which is deposited on the developing eggshell [14, 40]. For the marine aporocotylid species, *A. simplex*, we have provided morphological evidence for eggshell formation from modified vitelline globules in the form of a discontinuous, thin layer (0.07 μm in thickness) of electron-dense shell material around the fertilized ovum and associated vitellocytes [27]. Taking into account the fact that the eggshell of species of *Sanguinicola* and *Aporocotyle* is very thin [16, 18, 27, 32, 33, 38], this may explain the presence of a rather small number of globule clusters and globules within the clusters in their mature vitellocytes. The proportions of lipid droplets and glycogen in digenean vitelline cells, both of which represent nutritive material available to the developing embryo, vary between different digenean species [3, 11, 24, 25, 35, 36, 39]. Such differences may reflect variations in the retention of the eggs within host tissues or their development in the external environment, where they may survive for long periods waiting for transmission of the miracidium to the new host [2, 9, 10, 24, 35, 38]. In the case of *Sanguinicola*, Scheuring [33], Schell [32] and Kirk and Lewis [16] studied the life-cycle in *Sanguinicola* spp*.* and showed that eggs are released into the blood, carried to the branchial arteries and arterioles and ultimately become lodged in gill capillaries. Within seven days hatched miracidia pierce the capillary walls and the gill epithelium to be liberated into the environment, where they search out their molluscan intermediate host. It seems likely that most nutrients can pass through the thin eggshell while the eggs are developing in the blood vessels of the host.

This is the first study to examine the cytoarchitecture of the vitellarium of members of the freshwater lineage of aporocotylid blood-flukes. The study highlights differences in their vitelline cytoarchitecture compared with that of a member of the marine, teleost-infecting lineage of the Aporocotylidae. Since no member of the chondrichthyan-infecting lineage of the family, which likely occupy a more basal position in the aporocotylid tree [1], has been studied, it is not possible to assess the morpho-evolutionary steps in the formation of the aporocotylid vitellarium. However, the present data do appear to provide further morphological evidence supporting the divergent evolutionary lineages of marine and freshwater, teleost-infecting members of the family. The study supports our previous belief [25, 26] that the cytoarchitecture of the vitellarium represents an additional characteristic feature of the Digenea useful at family and lower taxonomic levels.

## Declaration of competing interest

The authors declare that they have no known competing financial interests or personal relationships that could have appeared to influence the work reported in this paper.
